# Perspectives on implementing Individual Placement and Support (IPS) within primary health care settings for adults living in British Columbia, Canada

**DOI:** 10.1186/s12888-023-05395-3

**Published:** 2023-12-07

**Authors:** Amanda Kwan, Stephany Berinstein, Jonathan Morris, Skye Barbic

**Affiliations:** 1 Department of Occupational Science and Occupational Therapy, Faculty of Medicine, University of British Columbia, Vancouver, BC Canada; 2 Canadian Mental Health Association BC Division, Vancouver, BC Canada; 3 Providence Research, Vancouver, BC Canada; 4 Centre for Health Evaluation Outcome Sciences, Vancouver, BC Canada

**Keywords:** Individual placement and support, Program implementation, Mental health, Vocational rehabilitation, Evidence-based practice, Community-based research

## Abstract

**Background:**

Individual placement and support (IPS) is an evidence-based practice (EBP) designed to help people with severe mental illness re-enter the labour market. Implementing an IPS program within a new context (e.g., primary health care setting) to support populations that are complex and multi-barriered presents a set of unique challenges and considerations. This paper provides community-based perspectives that identify implementation strengths and challenges and highlights potential strategies aimed at addressing emergent barriers.

**Methods:**

A case study was conducted across three community health centres in British Columbia (BC), Canada, where a novel IPS program was embedded within primary care services. Data collection consisted of open-ended surveys and focus groups with service providers directly involved in program implementation and their associated clinical and managerial support teams (*n* = 15). Using the updated Consolidated Framework for Implementation Research (CFIR) as a guide, we performed deductive thematic analysis to identify key areas impacting IPS implementation.

**Results:**

Integration with existing health care systems and primary health care teams and support from leadership across all levels were identified as both key facilitators and barriers to implementation. Facilitators and barriers were identified across all domains, with those within innovation and process most easily addressed. Four cross-cutting themes emerged for promoting more integrated and sustainable program implementation: investing in pre-implementation activities, supporting a dynamic and flexible program, building from community experiences, and developing a system for shared knowledge.

**Conclusions:**

Implementing an IPS program embedded within primary health care settings is complex and requires extensive planning and consultation with community-based service providers and decision-makers to achieve full integration. Future practice and policy decisions aimed at supporting employment and well-being should be made in collaboration with communities.

## Background

 Individual placement and support (IPS) is an evidence based practice (EBP) aimed at helping people with severe mental illness obtain mainstream or competitive employment [1]. EBPs are intended to improve the quality and efficiency of services for clients, with the goal of creating a positive shift in their outcomes [2, 3]. There is currently a large body of literature demonstrating that IPS produces better employment outcomes compared to traditional vocational services [4–7], with 55% of people with severe mental illness finding employment [8]. IPS programs have demonstrated improved outcomes across certain settings for other employment related outcomes (e.g., time to employment, employment length, hourly wage, number of hours), as well as non-employment outcomes (e.g., measures of quality of life) [4, 8]. Variability in these outcomes is associated with the degree to which a program adheres to the EBP model as it was originally designed, and the compatibility of the EBP with the target population.

Many EBPs embedded within a health setting fail during initial implementation, sustained implementation, or scaling-up because of implementation challenges [9]. This has fueled the field of implementation science and the development of implementation frameworks, theories, and models marked by one of three overarching aims: to describe implementation processes (process models), to explain implementation influences (determinant frameworks, classic theories, implementation theories), or to evaluate implementation (evaluation frameworks) [10]. In this paper, we focus on the aims of determinant frameworks, specifically using the updated Consolidated Framework for Implementation Research (CFIR) [11] for its broad and detailed consideration of contextual factors.

Known implementation challenges exist for health and social initiatives aimed at prevention, promotion, and/or intervention and often span multiple levels or domains, from individual and organizational characteristics to system-wide features. Each factor that holds influence can act as a facilitator towards successful implementation (strength) or a barrier impeding successful implementation (challenge), with factors often identified as both [12] based on different perspectives. Influencing factors also vary based on the EBP that is being implemented and the community-specific context surrounding it. Factors that are known to influence implementation include historical, cultural, and political context, health and social infrastructure, funding and resources, societal norms and stigma, organizational structure, climate, and readiness to change, along with knowledge, beliefs, and attitudes of service providers and clients [12–15]. Processes related to implementation, such as establishing protocols for communication, as well as the initiative or innovation itself, such as the degree to which it is compatible and adaptable for varying community contexts, also play a role in influencing the success of implementation [2, 14].

Known implementation challenges for IPS programs are centered around the compatibility and integration of IPS with existing services and systems providing employment support and/or social benefits, collaboration between IPS teams and different partners across mental health services, service provider or organizational resistance to change, and lack of security and funding to ensure sustainability [16–19]. IPS was originally developed in rural community mental health centres in the United States [20] but has since gained popularity worldwide. While implementation does not significantly differ in Canada [21], there are certain contextual differences (e.g., unique cultural factors, health systems and funding structures) that need to be considered to ensure appropriate implementation strategies are selected to address implementation barriers, specifically when integrating IPS into primary health care settings aimed at a more complex subpopulation. For example, in Canada, primary care teams increasingly have wraparound services, providing an opportunity to support both the health and social well-being of clients and their families. As primary health care settings are often the first point of contact for those seeking mental health supports, they have the potential to enhance the accessibility and effectiveness of employment services for individuals with mental illness. By integration of IPS within primary health care settings, for example within community health centres or other spaces where health and social services are co-located, opportunity exists to utilize a team-based or integrated approach to address both client health care needs and their employment goals [22]. There is currently little known about IPS implementation, including processes and facilitators/barriers, within this unique Canadian context.

Our overarching goal is to support the implementation of evidence-based IPS programs integrated into primary health care settings in British Columbia (BC), Canada. Specifically, through this paper we examined the following questions: *What are the factors influencing IPS implementation within primary health care settings and what actions can address prominent implementation barriers?* We explore the perspective of health care and social service providers to better understand community-driven solutions.

## Materials & methods

All components of this study were approved by the University of British Columbia Behavioural Research Ethics Board (BREB) on March 17, 2021 (UBC BREB# H20-02198). Informed consent was received from all study participants.

### Program design

**Fig. 1:**
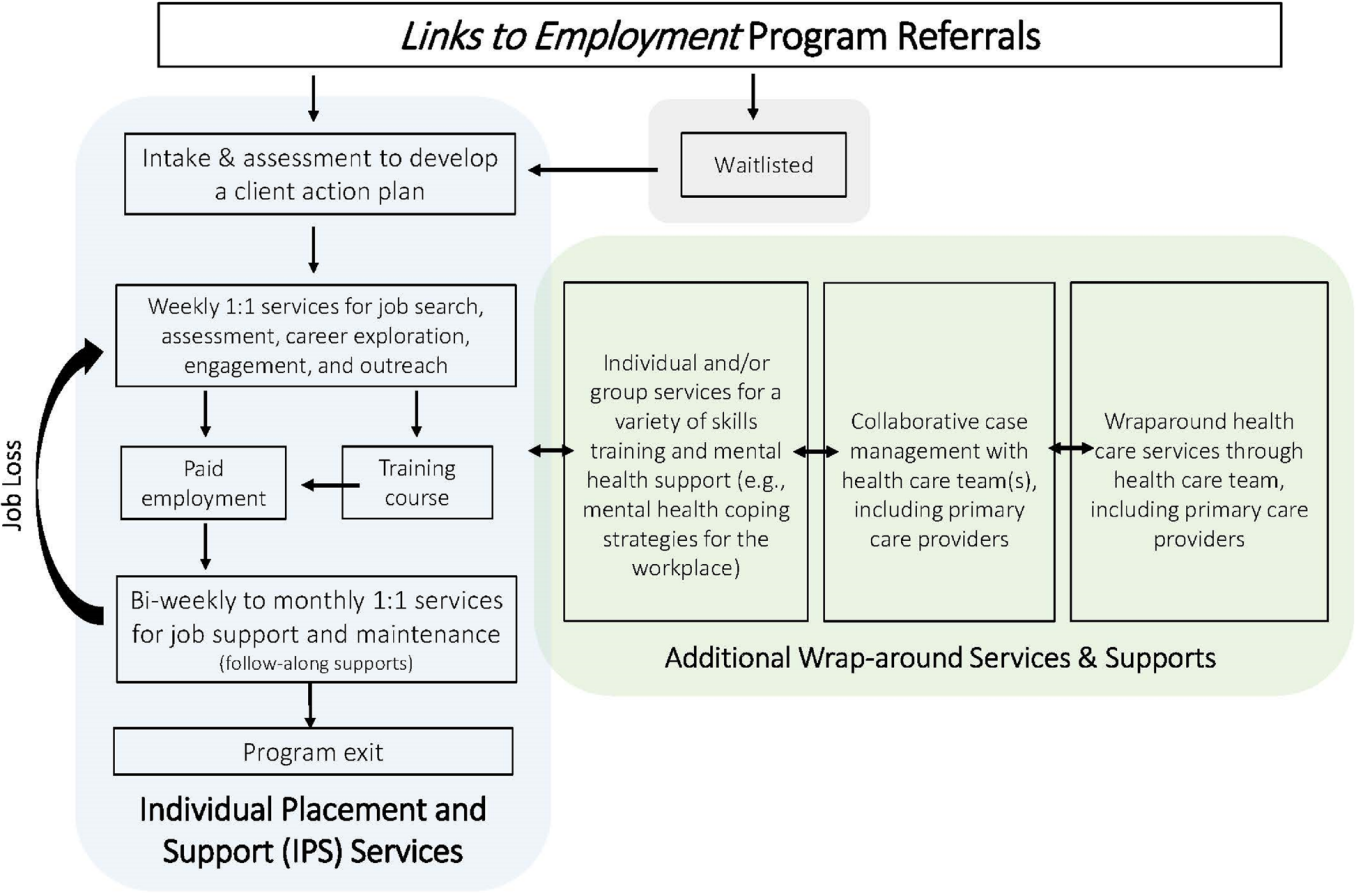
Program pathway

*Links to Employment* is a supported employment program, operating through the Canadian Mental Health Association BC Division (CMHA BC), aimed at supporting the employment goals of at-risk populations (e.g., those with severe mental illness and/or at risk of homelessness). The program is funded by the BC Ministry of Social Development and Poverty Reduction and based on the IPS model and guided by its eight principles: zero exclusion, competitive employment, mental health integration, benefits counseling, rapid job search, targeted job development, time unlimited, and individualized preferences [1]. *Links to Employment* is currently embedded within three community health centres (program sites), where primary health care services are available, in two BC communities (community sites). Within each community the program is delivered by vocational rehabilitation counsellors who have been trained in IPS through a combination of online courses (ipsworks.org) and internal educational sessions focused on practical application. The teams also include an occupational therapist(s) who conducts assessments to support vocational planning and provides clinical support and skills development through individual sessions and workshops. Additionally, the program teams work alongside a health care team, which includes integrated primary care providers who support crisis management and overall health maintenance of shared clients. Figure 1 below illustrates the intended program pathway that includes traditional IPS services as well as the wraparound health care services available.

### Study design

A multi methods study was conducted using open-ended surveys and focus group discussions with health care and social service providers.

### Study participants

Study participants consisted of service providers working directly and indirectly with the *Links to Employment* program. All health care and social service providers, including vocational rehabilitation counsellors, occupational therapists, social workers, and clinical and operational management directly involved with the program, were invited to participate in both data collection activities. Snowball sampling was then used to identify key individuals from associated clinical and managerial support teams across all participating community health centres. These individuals were invited to participate in a focus group and additionally asked to extend the invitation to key individuals within their own networks who may be impacted by or have impact on the program and its implementation. There were no exclusion criteria.

### Data collection

Open-ended surveys were administered to all program staff after one year of program implementation or at the end of their involvement with the program, whichever occurred first. To ensure that we captured the experience of all service providers who were involved with program implementation, we did not place a minimum time from which surveys could be administered. Of the surveys completed, the range of time was no less than 6 months, with most surveys completed in August 2022 at or after 1 year. The service provider survey covered topics related to service providers’ personal and professional experiences with IPS implementation and perspectives on the barriers and facilitators to supporting clients achieve their employment goals. Example questions included, *In your opinion, what key factors are needed to maximize the employment/education potential of Persons with Persistent Multiple Barriers (PPMB) over the long term?; How would you describe the implementation of this IPS program?;What were the key strengths/challenges around implementing this IPS program?*. The aggregate of these results were shared back during subsequent focus groups to guide wider discussion and consensus building, as well as included in the final analysis.

Second, a series of three focus groups were conducted between September – October 2022. Each focus group consisted of 4–7 individuals. Session #1 was tailored to those directly implementing the IPS program and involved semi-structured discussion around the experiences working with the priority population and implementing a new IPS program within primary care settings. The goal of the session was to build from the individual survey responses to collectively identify the range of implementation strengths and challenges or facilitators and barriers, including those that were perceived as key to successful implementation. Sessions #2 and #3 were inclusive of the wider team of health care and social service providers that either directly supported the program and/or worked alongside the same priority population. These sessions were used to validate the key strengths and challenges identified in Session #1, including clarifying and adding factors, as well as to discuss potential actions and adaptations to address key barriers. Each session was tailored to a specific community to allow for divergence based on unique contextual factors. Although participants across all focus groups were given a chance to individually respond to each facilitated question or prompt, the group was encouraged to interact and engage with one another to provide richer and more in depth discussion [23]. Example questions/prompts included, *Based on your experience with this program, are there other barriers or facilitators to implementation that haven’t been mentioned?; Discuss & identify the top 3 implementation challenges in your community; Is there anything about the implementation process that surprised you or didn’t surprise you?.* Each focus group was audio recorded and transcribed verbatim.

### Data analysis

All qualitative data were imported into NVivo 12 Pro data software managing tool. A deductive thematic analysis was employed using the domains and constructs outlined in the updated CFIR [11] as a guide. Not all constructs were represented in the focus group transcripts and emergent areas were not restricted to these categories. Analysis was performed in accordance with Braun & Clarke’s recommendations [24] to produce domain summaries and cross-cutting themes or areas for strategic action across all levels of influences on implementation.

## Results

A total of 15 service providers participated in this study, with 10 service providers directly involved with program implementation completing surveys and 11 service providers directly and indirectly involved with program implementation participating in focus groups. Table 1 below is a summary of participants, categorised by their role and whether they were internal or external to the program. Program externals were associated with the program and provided some aspect of program support (e.g., administrative, clinical) but were not directly involved in implementation. Given the sample size, participant demographics are not disclosed beyond role and level of involvement with implementation. The participation rate was 77% and 69%, respectively, for surveys and focus groups for those eligible and invited to participate.

**Table 1:** Summary of service provider participants

	Number Completed/In Attendance	
Role	Survey (*n* = 10)	Focus Group (*n* = 11)
Vocational Rehabilitation Counsellor/IPS specialist	5	4
Clinical Support/Management (program internal)	5	4
Clinical Support/Management (program external)	n/a	3

Through survey responses and focus groups, participants discussed the range of factors impacting their ability to delivery appropriate services to support the employment goals and well-being of clients. Participant perspectives are presented as domain summaries below with quotes to highlight their typical views.

### Innovation factors

The innovation in this study refers to the *Links to Employment* program or IPS program, as described in the “Program Design” section above. Participants identified that drawing from an evidence-based model supported implementation because others were familiar with IPS and confident in its effectiveness and approach (innovation source and evidence base), which helped give the program greater credibility and promote wider program buy-in. IPS principles also provided the implementation team with structure and guidance around best practice, especially when dealing with complex client cases or situations, although not all the principles consistently aligned with immediate client goals.

There’s other health authorities that are utilizing IPS – I had a great conversation with one of the directors of mental health and substance use and his [former workplace] had an IPS program in it. So, people know and people see the value.

### Outer setting factors

The outer setting in this study refers to both the wider health, social, and employment systems that provide programs, services, and supports for individuals across BC and the individual communities in which the IPS program was operationalized.

#### Financing

Insecure long-term funding was identified as a barrier that directly and indirectly impacted multiple aspects of program implementation, including staff turnover, health care service provider and organizational buy-in, and program integration into existing local systems of support.

We do feel like part of the primary care clinics now but having to qualify our work on whether we will continue or not because of funding, creates a barrier between us, our clinics, and our clients. To able to say we will support our clients through challenges long term, can give [them]the stability to trust us more.

#### Local conditions

Economic and environmental conditions were identified as a barrier impacting community-based engagement and support from local employers, as well as other agencies and organizations providing health or social services. Residual effects from the COVID-19 pandemic have created greater demand for services while capacity and human resources have simultaneously decreased, resulting in many businesses and organizations struggling to maintain current levels of operation.

[Our community] does have quite a lot of agencies and programs but I just think everything is so strained and challenged right now due to the current economic climate, the health care system … all of those factors. So that proves to be a big challenge.

Some of the challenges have been [around] making the local connections – having an employer that’s open and excited and wants to join the program and really have [that] community-based support.

### Inner setting factors

The inner setting in this study refers to the community health centres, which include primary health care services, where the IPS program is available, and the specific IPS program teams directly involved in implementation.

#### Information technology infrastructure

Being fully integrated into the system of electronic medical records within the primary care setting was identified as a facilitator towards promoting wider integration and collaborative care across both the program team and their associated health care service providers. An integrated system allowed both teams to independently stay up to date with all aspects of a client’s well-being, whether related to health or employment, allowing client needs to be met more quickly and with less repetition. Where the infrastructure was not in place to allow for a shared system of records, participants identified that it created multiple barriers.

Our lack of current integration with primary care is actually causing a barrier to clients because if they had a full network of support and integrated supports, then some of these challenges would be better solved or better supported and would just aid in [the] longevity of employment.

#### Work infrastructure

Staffing levels were identified as a continuous barrier to implementation. Not being able to find and/or retain highly qualified staff (e.g., occupational therapists) at each of the three program sites decreased program capacity and slowed program development. Gaps in staffing structure and supervision also limited the support available to the program team in areas such as overall program management and organization administration or reporting (e.g., waitlist management, funder reports, presentations), creating additional tasks and responsibilities outside the scope of employment-based service delivery.

More training and supervision was needed, especially in the early days of the program.

Participants also reported that staff turnover and capacity within their associated health care teams was a challenge, which often resulted in their clients being disconnected from health services or added to waitlists for health services. This required participants to shift their focus from traditional IPS or employment services to address critical health care needs.

There’s a lot of people in our community that need support and not a lot of resources for people to access at the moment. There’s been scope creep because there’s no support to refer people to in some cases.

#### Relationship connections

Although there was consensus around the importance of working relationships, there was a large disparity in relationship connections across the two levels of the inner setting and across both community sites. Working in collaboration, both through formal and informal pathways, with a health care team, which includes primary care providers, within the community health centre was identified as one of the key strengths supporting program implementation. An existing connection facilitated a more seamless transfer and sharing of clients between health and employment services and reinforced the important role that employment has in shaping an individual’s overall well-being. Additionally, being connected with a clinical team provided added support to clients with higher needs during times of immediate crises and for maintenance of overall health, which allowed program staff to focus more exclusively on employment related goals and work sustainment. Conversely, where there were not strong working relationships, integration was low or absent and IPS teams struggled to secure time with individual clinical staff to discuss shared clients and as a larger team to discuss program structure, referrals, and overall supports.

All referral sources should be from integrative health care teams; otherwise, clinical support and supervision is lacking.

Across the inner level of the inner setting (i.e., within the program team), relationship connections were consistently identified as a key strength for implementation. Built on shared values and working norms, the strong personal and professional relationships within the program team enabled sharing of difficult tasks, collective brainstorming, and collaborative troubleshooting and outreach. The team-based approach utilizes individual strengths to collectively support the diverse employment goals and well-being needs of clients across all program sites.

#### Culture (human equality and recipient-centredness)

Many of the guiding IPS principles, notably zero exclusion, time unlimited supports, individual preferences, and integration with mental health [25], were reported as aligning well with the needs of the population served, as well as with the existing client-centred values of each local community health centres and CMHA BC. This enabled the program to fit as a natural addition to existing services.

Focus[ing] on individualized support has been a great strength. The team has been able to work with clients who have a vast array of preferences and needs to develop goals that align with their desires.

### Individual factors

This domain highlights the roles and/or characteristics across various groups involved with the IPS program, as well as those that may impact or be impacted by its implementation.

#### High- and mid-level leaders

In this study, high-level leaders refer to those within the provincial government and/or provincial non-profits that hold decision-making power related to funding allocation and policies for health or social services and service delivery. Mid-level leaders refer to those responsible for making decisions and/or overseeing services at the local community level. Support at each of these levels was considered a vital component in securing buy-in to allow for the program to be operational and sustainable. Challenges at these levels created trickle-down implementation barriers for the IPS team.

If we could find the right person in the chain – I know we’ve had meetings with quite high-level health people, and they’re really enthusiastic … and we’ve had meetings with frontline staff, and they’re really enthusiastic. It’s sort of that mid mid-level management.

Integration into the clinics was also a long and arduous process that was greatly hindered by lack of communication from upper management.

#### Other implementation support

This role refers to all other health care service providers that support the program in various ways. Their support for the program was identified as the key factor for promoting integration between health and employment services, not only for incoming referrals, but also for continued communication and collaborative care. Where program support and buy-in from clinical teams was lacking, program staff struggled to build working relationships and ensure their clients had sufficient access to a range of other health resources.

[There’s] enthusiasm and support from partner clinics and the recognition of vocational rehabilitation as an important aspect of a person’s overall well-being. Clinicians have always been receptive and appreciative of the work that the team has been doing and are always open to consult with.

#### Implementation or program team

This role refers to those directly involved with implementing the IPS program and delivering IPS services to clients. Participants reported that the program team’s skills and experience, along with motivation and commitment to supporting clients, were critical components for successful implementation.

[There are] strong and diverse team members who are all devoted to serving the demographic we work with.

While most participants felt they had the tools and resources to implement the program, some reported inconsistent messaging and conflicting priorities across various levels of leaders and decision makers, including those internal and external to the program/community health centre. For example, during the early phases of implementation there was confusion around program requirements (e.g., inclusion criteria), service expectations (e.g., scope of services offered, IPS model), and operational logistics (e.g., where/how to document). Some participants also felt that additional training, especially for those without a clinical background, would be beneficial to ensure that complex client needs could be appropriately met.

The start to the program required education and courses on IPS, development of resources and program policies, and community networking. The time that it took to do these things were useful to ensure [there was] a solid base for the program to grow from.

There were many clients in our program with suicidal ideation. Additional training on this would be extremely necessary. Clients were much more complex and had much more trauma than we were prepared to address.

### Implementation process factors

In addition to sharing experiences and discussing the range of factors impacting program implementation, the focus group discussions provided opportunities for collective brainstorming around implementation strategies, suggested actions, and potential program adaptations to strengthen current implementation processes.

#### Assessing needs (program team)

Participants reported wanting clearer standards, guidelines, and tools for daily operation to increase consistency across messaging and operations and to minimize the time spent on administrative responsibilities. Where possible, participants wanted to reduce documentation and eliminate process redundancy. Participants also suggested developing an information resource that would provide a current list of available health programs, services, and supports within their community to ensure access to the existing network of health services. This type of resource would include local services offered within each community health centre (e.g., pain management, substance use support), as well as those available at the provincial level.

I had a lot of trouble directing [clients] to a place within primary care. Kind of like if they had addictions issues, directing them to a place where they can be supported – it’s not just the need to integrate, it’s also navigation, help navigating to specific services.

Lastly, participants noted that their perspectives and opinions should have greater weight in the decisions made around program practices and policy. Many reporting feeling that routinely incorporating service provider feedback and moving towards a bottom-up leadership structure could benefit program design, ensure ongoing fit with client needs, and support timely solutions to address implementation challenges.

#### Assessing needs (program clients)

Participants suggested additional program-based wraparound supports to help meet the complex and dynamic needs of the program clients. Specific suggestions included adding peer support and opportunities for more community connection, workshops focused on basic life and job skills (e.g., money management, basic computer literacy, communication), and integration of other psychosocial rehabilitation practices aimed at promoting recovery and wellness. Additionally, participants suggested greater integration and use of allied health support. In BC, the allied health workforce consist of multiple regulated health providers such as dieticians, social workers, massage therapists, music therapists, kinesiologists, etc., that provide a range of preventative, diagnostic, technical, and therapeutic health services considered outside the scope of primary health care [26].

Client cases are quite complex and require much more support outside of vocational activities – Clients often require outside referrals and resources to support their day-to-day.

#### Tailoring strategies

Participants identified the need for different program sites to focus on different implementation strategies based on their unique barriers. For sites struggling with referrals and program integration, participants suggested increasing visibility to spread awareness and promote the program. Suggested strategies included developing program advertisements (e.g., brochures, posters) and securing co-location within a dedicated space. In addition, frequent opportunities to share client outcomes and successes with broader clinical teams would highlight the value of the program to overall client well-being, helping to foster relationship connections across various partners and levels of management, particularly those identified as mid-level leaders.

Continuing just to connect as much as possible, to go over to that site – I think that lack of presence is a real contributing factor to the challenges that the team is experiencing.

For sites struggling to build community-based employer relationships, participants suggested ongoing resources and educational opportunities for employers to help address stigma in the workplace and to create an inclusive and flexible working environment (e.g., creating lower barrier jobs to meet hiring demands).

Support for employers to know how to hire and accommodate those with multiple barriers.

#### Engaging (program team)

Participants suggested options to build existing staff and team capacity and capability by providing additional resources, training, and supports. In addition, participants felt that the program team should include an occupational therapist at each site and an integrated counsellor at each community health centre dedicated specifically to program clients. This would ensure low-barrier and consistent access to mental health services in addition to primary care services.

#### Adapting

Participants identified the need for program flexibility as a key component for supporting ongoing implementation. This included extending the scope of services provided beyond traditional IPS to help support clients in other areas of their life that would bolster transferable skills and indirectly increase work readiness. For example, including peer support opportunities for being mentored and/or offering mentorship.

A lot of the time that mental health aspect is lacking because a lot of people have become quite isolated and then they actually don’t have that peer connection and that can be a limiting factor, so maybe expanding that peer support.

Participants also discussed adapting core IPS principles to better meet the specific needs of their clients. For example, where IPS exclusively promotes competitive employment and discourages the use of sheltered or temporary work placements [27], participants described clients with higher needs were interested in volunteering or engaging in temporary work placements to build confidence and gain “low-risk” experience.

Some sort of work experience placement that could help with community buy-in with employers, while at the same time, providing clients who maybe don’t have education or experience with real experience [to] you know, help them with some skills. And also provide references and things like that for jobs that they want to do in the future.

Implementation barriers are present in all five CFIR domains described above. Barriers within the innovation and process domains were identified as easiest to address; however, there was recognition of the importance of addressing key barriers and/or influential barriers, for example those centered on upstream factors.

## Discussion

The aim of this study was to determine community-based perspectives around how different factors influence implementation of an IPS program integrated within primary health care settings and identify cross-domain strategies that might mitigate implementation challenges being experienced. Analysis across domains identified four emergent cross-domain themes or strategies based on service provider experiences: the need to invest more in pre-implementation activities, support a dynamic and flexible program, build from community experiences, and develop a system for shared learnings to support successful implementation. Each cross-cutting strategy or theme is discussed below.

### Invest in pre-implementation activities

Although many pre-implementation activities occurred (e.g., a problem/service gap was identified, funding for a new program was secured, high level stakeholders were engaged for program operations and evaluation), there were necessary components at this stage that were underdeveloped or incorporated ad hoc during the implementation stage, most critically consistent engagement and buy-in from mid-level leaders. During pre-implementation, all relevant partners and stakeholders should be engaged and share an understanding of the responsibilities and resources required for successful implementation [2]. Leadership plays an important role in defining organizational context and setting cultural norms [28] that can mitigate or contribute to common inner setting barriers, such as clinical engagement, organizational resistance to change, and cooperation between employment teams (program team) and health care teams [29, 30]. More time and resources should be allocated to securing early buy-in from all levels of leadership when implementing any IPS program, but it is especially important given the inter-sectoral space of the *Links to Employment* program and the potential difference in priorities of employment and primary care services.

In addition to this, pre-implementation activities should also focus on defining expectations and developing a process for clear communication within and across teams. While these findings are consistent with supporting integration of IPS with mental health services [31], we emphasize the necessity of these processes during pre-implementation before program clients are involved, and across all relevant health services. Not only would this strengthen individual and team capabilities but it could help prevent known barriers related to knowledge gaps (e.g., sufficient understanding of IPS) and resulting negative attitudes or beliefs [18, 32] that subsequently impact relationship connections during implementation.

### Support a dynamic and flexible program

To support strict adherence to the IPS model, the eight core principles are further broken down into 25 fidelity items that provide specific guidance around goals for quality improvement across the areas of staffing, organization, and services [27]. Flexibility and adaptations from these are not encouraged under the standard IPS model; however, the complexity and range of barriers beyond mental health challenges experienced by our program’s priority population requires a more enhanced and dynamic approach, with sustained funding to support those that need ongoing IPS engagement over time. Adherence to EBP models developed for other populations are not sufficient for supporting all sociodemographic groups or considering the cultural or contextual differences that may exist [33]. In practice, supported employment programs across Canada have high variability in different measures of fidelity and program priority [21] as a result of local program adaptations. While adaptations may reduce program effectiveness [34, 35], there is limited evidence as to which components of IPS are essential and how certain adaptations might impact employment outcomes for complex adult populations who concurrently experience a range of barriers in a Canadian setting (e.g., unique cultural factors, drug toxicity crisis, funding structures). There is a growing body of literature examining IPS in comparison to other interventions or combinations of interventions (e.g., augmented supported employment, pre-vocational training, transitional employment, enhanced IPS) [7, 36] beyond traditional vocational services, and focusing on additional program enhancements that may provide a more matched and tailored level of support for those with varying needs [37]. This study supports the need to explore IPS adaptation and enhancements, specifically for complex populations that have been underrepresented in the employment space and employment research. Flexible implementation processes, guided by the needs of program clients and service providers, could result in more targeted and wraparound services specific for a population with complex needs, which could result in higher health and employment outcomes over time.

### Build from community expertise

Poor implementation and implementation challenges contribute to the failure of many complex and expensive innovations embedded within health care settings [9]. A known solution to this is to build underlying support for new EBP through strong, engaged partnerships that involve active participation and community-led or shared decision-making across all individuals/organizations that may impact or be impacted by implementation [14]. One example is adopting an implementation process that not only routinely assesses service provider and client needs but also includes a formal and iterative process where their feedback and suggestions on current practices or processes can be trialed within the program. Engaging frontline service providers, including relevant clinicians and health care teams, is essential to ensure that the program itself, as well as implementation decisions and strategies, are suitable to the existing local context [2, 14]. Given the rigidity of IPS implementation when adhering with fidelity practices, a community-based participatory research approach has not been widely considered by IPS programs, despite having the potential to strengthen local ownership of community-based initiatives, such as the *Links to Employment* program, thereby increasing program support and future sustainability [38, 39].

### Develop a system for shared and sharing knowledge

The final emergent theme for promoting a more integrated and sustainable program involves developing a system for sharing knowledge that enables multiple programs sites to learn from themselves and their shared network. This is especially important as the *Links to Employment* program scales to include more sites across the province. A learning health system (LHS), defined as a system where *“science, informatics, incentives, and culture are aligned for continuous improvement and innovation, with best practices seamlessly embedded in the delivery process and new knowledge captured as an integral by-product of the delivery experience”* [40], would support a more effective approach to program implementation, program adaptation, and localized service delivery by drawing from continuous and collective data. Access to real-time data and its interpretation could also promote greater buy-in across individuals and organizations by supporting rapid dissemination of client success stories and program-level metrics that showcase the added value of the program towards overall client well-being. Development of a LHS within an IPS program embedded within primary health care settings would be a novel addition to the existing systems of health and employment support in BC.

### Study limitations

This study provides novel insights into the strengths and challenges associated with implementing an IPS program within a primary health care setting, however, there are study limitations to be noted. First, our sample size was small given the current scope of the program, and we did not have representation from all external health care teams due to conflicting schedules and capacity. We also did not engage decision makers or leadership at every level. Greater representation across all primary health care settings would ensure that unique contextual factors are being sufficiently considered. Second, our study was focused on the perspectives of service providers during the early phases of implementation an EBP. Ongoing research is needed to understand additional facilitators and barriers across different phases of implementation, including during iterative cycles of adaptation and re-design, and towards processes that enable long-term sustainment of service delivery and funding.

## Conclusion

Our objective was to understand service provider perspectives on the factors that facilitate and impede program implementation within a multi-sector context and identify potential strategies towards more integrated and sustainable service for adult populations with complex health and social needs. The *Links to Employment* program provides an example of how employment and primary health care services can complement one another in a real-world setting and the range of factors that should be considered. Further research should move towards building a more participatory process with service providers positioned to guide practice and policy decisions impacting implementation.

## Data Availability

The datasets generated and/or analysed during the current study are not publicly available due to privacy or ethical restrictions but are available from the corresponding author on reasonable request.

## Abbreviations

EBP: Evidence–based practice
CFIR: Consolidated Framework for Implementation Research
IPS: Individual Placement and Support
BC: British Columbia
CMHA BC: Canadian Mental Health Association British Columbia Division
LHS: Learning Health System

## References

[1] Bond GR (2004). Supported employment: evidence for an evidence-based practice. Psychiatr Rehabil J.

[2] Kilbourne AM, Goodrich DE, Miake-Lye I, Braganza MZ, Bowersox NW (2019). Quality enhancement research initiative implementation roadmap: toward sustainability of evidence-based practices in a learning health system. Med Care.

[3] Tucker S, McNett M, Mazurek Melnyk B, Hanrahan K, Hunter SC, Kim B (2021). Implementation science: application of evidence-based practice models to improve healthcare quality. Worldviews Evid Based Nurs.

[4] Frederick DE, VanderWeele TJ. Supported employment: meta-analysis and review of randomized controlled trials of individual placement and support. PLoS ONE. 2019;14(2):e0212208. 10.1371/journal.pone.0212208 PMC6382127 30785954

[5] Kinoshita Y, Furukawa TA, Kinoshita K, Honyashiki M, Omori IM, Marshall M et al. Supported employment for adults with severe mental illness. Cochrane Database Syst Rev. 2013;(9). Available from: https://www.readcube.com/articles/10.1002%2F14651858.CD008297.pub2. [Cited 2021 Apr 13]. 10.1002/14651858.CD008297.pub2 PMC7433300 24030739

[6] Modini M, Tan L, Brinchmann B, Wang MJ, Killackey E, Glozier N (2016). Supported employment for people with severe mental Illness: systematic review and meta-analysis of the international evidence. Br J Psychiatry.

[7] Suijkerbuijk YB, Schaafsma FG, van Mechelen JC, Ojajärvi A, Corbière M, Anema JR (2017). Interventions for obtaining and maintaining employment in adults with severe mental Illness, a network meta-analysis. Cochrane Database Syst Rev.

[8] Bond GR. Evidence for the Effectiveness of Individual Placement and Support Model of Supported Employment. 2022. Available from: https://docs.google.com/presentation/d/1RFqFrzidP_EwUEb_tgZ57LUJGpodbCM-7oQIQDda1P8. [Cited 2023 Jan 30].

[9] Jacobs SR, Weiner BJ, Reeve BB, Hofmann DA, Christian M, Weinberger M (2015). Determining the predictors of innovation implementation in healthcare: a quantitative analysis of implementation effectiveness. BMC Health Serv Res.

[10] Nilsen P (2015). Making sense of implementation theories, models and frameworks. Implement Sci IS.

[11] Damschroder LJ, Reardon CM, Widerquist MAO, Lowery J (2022). The updated consolidated framework for implementation research based on user feedback. Implement Sci.

[12] Le PD, Eschliman EL, Grivel MM, Tang J, Cho YG, Yang X (2022). Barriers and facilitators to implementation of evidence-based task-sharing mental health interventions in low- and middle-income countries: a systematic review using implementation science frameworks. Implement Sci.

[13] Aarons GA, Green AE, Trott E, Willging CE, Torres EM, Ehrhart MG (2016). The roles of system and organizational leadership in system-wide evidence-based intervention sustainment: a mixed-method study. Adm Policy Ment Health Ment Health Serv Res.

[14] Durlak JA, DuPre EP (2008). Implementation matters: a review of research on the influence of implementation on program outcomes and the factors affecting implementation. Am J Community Psychol.

[15] Kelly P, Hegarty J, Barry J, Dyer KR, Horgan A (2017). A systematic review of the relationship between staff perceptions of organizational readiness to change and the process of innovation adoption in substance misuse treatment programs. J Subst Abuse Treat.

[16] Bergmark M, Bejerholm U, Markström U (2018). Critical components in implementing evidence-based practice: a multiple case study of individual placement and support for people with psychiatric disabilities. Soc Policy Adm.

[17] Hasson H, Andersson M, Bejerholm U. Barriers in implementation of evidence-based practice: Supported employment in Swedish context. Dickinson H, Millar R, West M, editors. J Health Organ Manag. 2011;25(3):332–45. 10.1108/14777261111143563 21845986

[18] Vukadin M, Schaafsma FG, Westerman MJ, Michon HWC, Anema JR (2018). Experiences with the implementation of Individual Placement and Support for people with severe mental illness: a qualitative study among stakeholders. BMC Psychiatry.

[19] Latimer E, Bordeleau F, Méthot C, Barrie T, Ferkranus A, Lurie S (2020). Implementation of supported employment in the context of a national Canadian program: facilitators, barriers and strategies. Psychiatr Rehabil J.

[20] Bond GR, Lockett H, van Weeghel J. International growth of individual placement and support. Epidemiol Psychiatr Sci. 2020 ed ;29. Available from: https://www.cambridge.org/core/journals/epidemiology-and-psychiatric-sciences/article/international-growth-of-individual-placement-and-support/C48BB5880D1CD8C0DB69A8CC7D68A68B. Cited 2021 Mar 16. 10.1017/S2045796020000955 PMC7681150 33185176

[21] Corbière M, Bond GR, Goldner EM, Ptasinski T (2005). Brief reports: the Fidelity of supported employment implementation in Canada and the United States. Psychiatr Serv.

[22] Premier O. of the. B.C. government’s primary health-care strategy focuses on faster, team-based care | BC Gov News. 2018. Available from: https://news.gov.bc.ca/releases/2018PREM0034-001010. Cited 2023 Jul 31.

[23] Krueger R, Casey M. Focus Groups: A Practical Guide for Applied Research. 5th ed. SAGE Publications Inc; 2014. Available from: https://us.sagepub.com/en-us/nam/focus-groups/book243860.

[24] Braun V, Clarke V (2019). Reflecting on reflexive thematic analysis. Qual Res Sport Exerc Health.

[25] Swanson SJ, Becker DR (2013). IPS supported employment: a practical guide.

[26] enews-allied-health-. summer-2022.pdf. Available from: https://www2.gov.bc.ca/assets/gov/health/about-bc-s-health-care-system/heath-care-partners/health-newsletter/enews-allied-health-summer-2022.pdf. Cited 2023 Jul 31.

[27] Becker DR, Swanson S, Bond GR, et al. Evidence-based supported employment fidelity review manual. Lebanon: Dartmouth Psychiatric Research Center; 2008.

[28] Aarons GA, Ehrhart MG, Farahnak LR, Sklar M (2014). Aligning Leadership across systems and organizations to develop a strategic climate for evidence-based practice implementation. Annu Rev Public Health.

[29] Bergmark M, Bejerholm U, Markström U (2019). Implementation of evidence-based interventions: analyzing critical components for sustainability in community mental health services. Soc Work Ment Health.

[30] Bonfils IS, Hansen H, Dalum HS, Eplov LF (2017). Implementation of the individual placement and support approach – facilitators and barriers. Scand J Disabil Res.

[31] Bond GR, Becker DR, Drake RE, Rapp CA, Meisler N, Lehman AF (2001). Implementing supported employment as an evidence-based practice. Psychiatr Serv Wash DC.

[32] Handler J, Doel K, Henry A, Lucca A (2003). Rehab rounds: implementing supported employment services in a Real-World setting. Psychiatr Serv.

[33] Goldman HH, Ganju V, Drake RE, Gorman P, Hogan M, Hyde PS (2001). Policy implications for implementing evidence-based practices. Psychiatr Serv.

[34] Becker DR, Smith J, Tanzman B, Drake RE, Tremblay T (2001). Fidelity of supported employment programs and employment outcomes. Psychiatr Serv Wash DC.

[35] McCrabb S, Lane C, Hall A, Milat A, Bauman A, Sutherland R (2019). Scaling-up evidence-based obesity interventions: a systematic review assessing intervention adaptations and effectiveness and quantifying the scale-up penalty. Obes Rev.

[36] Prior S, Maciver D, Aas RW, Kirsh B, Lexen A, van Niekerk L (2020). An enhanced individual placement and support (IPS) intervention based on the Model of Human Occupation (MOHO); a prospective cohort study. BMC Psychiatry.

[37] Dewa CS, Loong D, Trojanowski L, Bonato S (2018). The effectiveness of augmented versus standard individual placement and support programs in terms of employment: a systematic literature review. J Ment Health.

[38] Cashman SB, Adeky S, Allen AJ, Corburn J, Israel BA, Montaño J (2008). The power and the promise: working with communities to analyze data, interpret findings, and get to outcomes. Am J Public Health.

[39] Wallerstein N, Duran B (2010). Community-based participatory research contributions to intervention research: the intersection of science and practice to improve health equity. Am J Public Health.

[40] Care I, of M (US) R on V&, Yong SDH, Olsen PL, McGinnis L. JM. Institute of Medicine: Roundtable on Value & Science-Driven Health Care: Charter and Vision Statement. In: Value in Health Care: Accounting for Cost, Quality, Safety, Outcomes, and Innovation. National Academies Press (US); 2010 . Available from: https://www.ncbi.nlm.nih.gov/books/NBK50934/. Cited 2023 Jul 4. 21210558

